# 30-Day Outcomes of Simultaneous Sleeve Gastrectomy and Kidney Transplantation: An Analysis from the Metabolic and Bariatric Surgery Accreditation and Quality Improvement Program (MBSAQIP) Database

**DOI:** 10.1007/s11695-026-08815-x

**Published:** 2026-06-27

**Authors:** Doua Elamin, Valentin Mocanu, Mélissa V. Wills, Andrew Strong, Salvador Navarrete, Ricard Corcelles, Matthew Kroh, Jerry Dang

**Affiliations:** Cleveland Clinic, Cleveland, USA

**Keywords:** Sleeve gastrectomy, Kidney transplantation, Bariatric surgery, Metabolic and bariatric surgery accreditation and quality improvement program (MBSAQIP), End-stage renal disease (ESRD), Simultaneous surgery

## Abstract

**Background:**

Obesity limits access to kidney transplantation (KT) and worsens outcomes in patients with end-stage renal disease (ESRD). Sleeve gastrectomy (SG) is increasingly used to facilitate transplant eligibility; however, the safety of simultaneous SG and KT (SG + KT) remains poorly defined at a multi-institutional level. This study evaluates 30-day perioperative outcomes of SG + KT using the Metabolic and Bariatric Surgery Accreditation and Quality Improvement Program (MBSAQIP) database.

**Methods:**

A retrospective analysis of the MBSAQIP database (2020–2023) was performed. Adult patients undergoing SG were identified with simultaneous KT and captured using CPT codes. Demographics, comorbidities, operative characteristics, and 30-day outcomes were compared between SG-only and SG + KT cohorts. Multivariable logistic regression was used to identify independent predictors of serious complications and 30-day mortality.

**Results:**

Among 582,860 patients undergoing SG, 22 (0.004%) underwent simultaneous KT. SG + KT patients were older, predominantly male, and had significantly higher rates of diabetes, hypertension, renal insufficiency, and dialysis dependence (all *p* < 0.001), with similar body mass index compared to SG alone. SG + KT was associated with longer operative time, increased length of stay, and higher rates of bleeding, readmission within 30-days and serious complications (all *p* ≤ 0.001). Thirty-day mortality was significantly higher following SG + KT (9.1% vs. 0.1%, *p* < 0.001). On multivariable analysis, simultaneous KT was the strongest independent predictor of serious complications (OR 7.97; 95% CI 2.27–27.93; *p* = 0.001) and mortality (OR 33.99; 95% CI 3.99–289.03; *p* = 0.001).

**Conclusion:**

Simultaneous sleeve gastrectomy and kidney transplantation is exceedingly rare and associated with markedly increased 30-day morbidity and mortality compared with sleeve gastrectomy alone, highlighting the need for careful patient selection and multidisciplinary risk assessment.

## Key Points

Simultaneous sleeve gastrectomy and kidney transplantation occur in 0.004% of bariatric cases.

Combined procedures are associated with significantly increased 30-day complications and mortality.

Simultaneous kidney transplantation is the strongest independent predictor of postoperative death.

## Introduction

Obesity remains a global public health crisis. Recent modeling studies project that, if current trends continue, the prevalence of adult obesity in the U.S. will approach 49% by 2030, with severe obesity affecting nearly one in four adults [1]. Its implications are particularly profound in patients with end-stage renal disease (ESRD), where it exacerbates comorbid conditions and poses significant challenges to kidney transplantation (KT) eligibility and outcomes [2]. Obesity is also associated with a significantly increased risk of chronic kidney disease (CKD) progression [3]. 

People with obesity are often excluded from transplant waitlists due to elevated perioperative risk, wound complications, delayed graft function, and increased mortality. The Kidney Disease: Improving Global Outcomes (KDIGO) guideline recognizes obesity as a relative contraindication to kidney transplantation and notes that patients with class II or III obesity (Body Mass Index (BMI) ≥ 35 kg/m²) should be considered for interventions such as dietary counseling or bariatric surgery, and transplantation in patients with BMI ≥ 40 kg/m² should be approached with caution due to increased postoperative complications and mortality risk [4]. 

Bariatric surgery, especially sleeve gastrectomy (SG), has emerged as a viable strategy to improve access to transplantation due to resolution of obesity and associated metabolic comorbidities [5, 6]. The timing and sequencing of bariatric surgery in relation to transplantation is an area of investigation. Pre-transplant bariatric surgery was found to improve access to KT in patients with severe obesity. Transplant recipients also continued to benefit from sustained weight loss and improved related comorbidities that may positively impact their graft function after KT [7]. In a study comparing bariatric surgery (BS) pre vs. post KT, no differences in the main outcomes were identified, but BS post KT group tended to gain more weight during follow-up [8]. Another study noted that after KT, BS-induced weight loss appeared to positively impact the function of the grafted kidney, but increased graft-rejection risk, possibly because of changes in the bioavailability of immunosuppressant drugs [9]. 

However, simultaneous sleeve gastrectomy and kidney transplantation (SG + KT) has recently emerged as an innovative approach to address the limitations of staged procedures. In a single-center retrospective evaluation of kidney transplant recipients, simultaneous kidney transplant and sleeve gastrectomy resulted in successful weight loss compared to kidney transplant alone, while maintaining similar rates of graft and patient survival [10]. Nevertheless, robust data on perioperative outcomes, particularly from multi-institutional sources, remain scarce, potentially limiting global adoption. As the integration of metabolic and transplant surgery gains momentum, there is a critical need to evaluate outcomes at a broader scale. To address this gap, the current study leverages the Metabolic and Bariatric Surgery Accreditation and Quality Improvement Program (MBSAQIP) database to produce the first comprehensive multi-centered international analysis to assess 30-day perioperative outcomes in patients undergoing SG + KT.

## Materials and Methods

A retrospective analysis was performed utilizing files from the MBSAQIP database from 2020 to 2023 as key variables relevant to the analysis, particularly those related to renal disease severity and perioperative risk factors were standardized and consistently captured in MBSAQIP starting in 2020. The MBSAQIP is a national registry that collects standardized, clinical data from over 900 bariatric surgery centers across North America. It includes detailed perioperative variables and 30-day outcomes for patients undergoing bariatric surgery. The use of de-identified data exempted this study from Institutional Review Board (IRB) review.

### Patient Selection

We identified all adult patients who underwent SG using CPT code 43,775. Patients undergoing KT were identified by searching for CPT codes: 50,360, 50,365, 50,370, and 50,380. Only elective cases were included. Patients with CPT codes indicating Roux-en-Y gastric bypass or other bariatric revisions, emergency, or endoscopic bariatric procedures were excluded. After identifying all KT cases, we created two separate cohorts: SG + KT and SG.

### Pre-intra- and Post-operative Variable Definitions

Basic Demographic variables including age, sex, race, and body mass index (BMI) were collected. Metabolic comorbidities included hypertension, diabetes (insulin and non-insulin dependent), dialysis, sleep apnea, history of venous thromboembolism (VTE), and renal insufficiency. Functional status variables included American Society of Anesthesiologists (ASA) classification. Operative variables included surgical approach (laparoscopic, open, or endoscopic), operative time, and length of stay. Other clinical variables included albumin level and therapeutic anticoagulation. Serious complications is a composite variable defined by the investigators based on standard conventions used in surgical outcomes research, including the ACS risk calculator. This composite is defined by the presence of deep surgical site infection, VTE, sepsis, reoperation, pneumonia, myocardial infarction, unplanned intubation, or ICU admission. This composite outcome includes complications not individually reported in outcome tables.

The primary outcomes were to identify the prevalence of SG + KT as well as to characterize differences between cohorts. Secondary outcomes included evaluating differences between 30-day morbidity and mortality.

### Statistical Analysis

Continuous variables were described as absolute values +/- standard deviation and evaluated using Wilcoxon rank-sum tests or t-tests. Categorical variables were described as percentages and absolute values and analyzed using chi-square or Fisher’s exact tests. Differences between the SG-only group and those undergoing SG + KT were assessed using univariate logistic regression. Multivariable logistic regression was used to identify predictors of serious complications and 30-day mortality. The model incorporated patients’ baseline characteristics and surgical variables. Dialysis status was not included in the final multivariable model due to collinearity with renal insufficiency and simultaneous SG + KT variable, as most SG + KT patients were dialysis dependent. Collinearity was assessed using variance inflation factors (VIFs). Model discrimination and calibration were evaluated using the area under the receiver operating characteristic (AUROC) curve and the Brier score. All statistical analyses were conducted using Stata version 17 (StataCorp, College Station, TX, USA).

## Results

### Patient Characteristics

Between 2020 and 2023, a total of 582,860 patients underwent SG as recorded in the MBSAQIP database. Of these, 22 patients (0.004%) underwent SG + KT. Patients undergoing SG + KT were significantly older than those undergoing SG alone (mean age 49.5 ± 8.4 vs. 43.2 ± 11.9 years, *p* = 0.01), and were more likely to be male (54.6% vs. 18.2%, *p* < 0.001) with no differences in BMI between groups (43.5 ± 6.4 vs. 44.8 ± 8.0 kg/m², *p* = 0.47). SG + KT patients were significantly more likely to have diabetes (68.2% vs. 20.4%, *p* < 0.001), hypertension (86.4% vs. 42.2%, *p* < 0.001), and renal insufficiency (77.3% vs. 0.5%, *p* < 0.001). The majority of SG + KT cohort (63.6%) required dialysis at the time of surgery, compared to 0.3% in the SG group (*p* < 0.001). The open surgical approach was used more frequently in SG + KT cases (9.1% vs. 0.2%, *p* < 0.001) associated with a longer operative time (330.2 ± 107.9 vs. 75.0 ± 44.8 min, *p* < 0.001) and an increased median postoperative length of stay (8.6 vs. 1.2 days, *p* < 0.001) (Table 1).

**Table 1:** Patient characteristics and operative details

Characteristic	SG (*n* = 582,838)	SG + KT (*n* = 22)	*P*-value
Age, years (mean ± SD)	43.2 ± 11.9	49.5 ± 8.4	0.01
BMI, kg/m² (mean ± SD)	44.8 ± 8.0	43.5 ± 6.4	0.47
Male sex n (%)	106,032 (18.2)	12 (54.6)	< 0.001
Diabetes n (%)	119,161 (20.4)	15 (68.2)	< 0.001
Hypertension n (%)	246,240 (42.2)	19 (86.4)	< 0.001
Dialysis dependence n (%)	1,951 (0.3)	14 (63.6)	< 0.001
Renal insufficiency n (%)	2,995 (0.5)	17 (77.3)	< 0.001
Albumin, g/dL (mean ± SD)	4.15 ± 0.40	3.74 ± 0.56	0.004
ASA Class III–IV (%)	466,623 (80.1)	22 (100.0)	< 0.001
Therapeutic anticoagulation n (%)	16,794 (2.9)	1 (4.6)	0.64
Surgical approach: Open n (%)	1,387 (0.2)	2 (9.1)	< 0.001
Operative time, min (mean ± SD)	75.0 ± 44.8	330.2 ± 107.9	< 0.001
Length of stay, days (mean ± SD)	1.2 ± 1.2	8.6 ± 4.3	< 0.001

### Operative Outcomes and Complications

Patients undergoing SG + KT had significantly higher rates of serious complications within 30 days compared to SG alone (22.7% vs. 2.5%, *p* < 0.001). Specifically, rates of postoperative bleeding (13.6% vs. 0.7%, *p* < 0.001), readmission (31.8% vs. 2.5%, *p* < 0.001) and venous thromboembolism (4.5% vs. 0.4%, *p* = 0.002) were significantly increased. There were no significant differences in rates of staple line leak (0.0% vs. 0.3%, *p* = 0.81), reoperation (0.0% vs. 0.8%, *p* = 0.67), or interventional procedures (0.0% vs. 0.6%, *p* = 0.71). 30-day mortality was significantly higher among SG + KT patients (9.1% vs. 0.1%, *p* < 0.001) (Table 2). The mortality rate of ESRD patients on dialysis undergoing SG alone was found to be 0.81%. While MBSAQIP does not capture transplant waitlist status or eligibility, this subgroup represents the closest available comparator to outcomes in patients with advanced renal disease.

**Table 2:** Postoperative outcomes of sleeve gastrectomy (SG) with and without simultaneous kidney transplantation (KT)

Outcome	SG (*n* = 582,838)	SG + KT (*n* = 22)	*P*-value
Serious complications n (%)	14,299 (2.5)	5 (22.7)	< 0.001
Bleeding n (%)	4,279 (0.7)	3 (13.6)	< 0.001
Readmission within 30 days n (%)	14,759 (2.5)	7 (31.8)	< 0.001
Venous thromboembolism n (%)	2,253 (0.4)	1 (4.5)	0.002
Reoperation within 30 days n (%)	4,754 (0.8)	0 (0.0)	0.67
Intervention within 30 days n (%)	3,515 (0.6)	0 (0.0)	0.71
Staple line leak n (%)	1,539 (0.3)	0 (0.0)	0.81
30-day mortality n (%)	395 (0.1)	2 (9.1)	< 0.001

### Multivariable Analysis

On multivariable logistic regression analysis, simultaneous kidney transplantation was the strongest independent predictor of 30-day serious complications (OR 7.97; 95% CI: 2.27–27.93; *p* = 0.001) after adjusting for age, sex, BMI, diabetes, hypertension, renal insufficiency, myocardial infarction history, albumin, and anticoagulation status. Similarly, SG + KT was associated with a 33.99-fold increase in odds of 30-day mortality (OR 33.99; 95% CI: 3.99–289.03; *p* = 0.001), independent of other clinical covariates (Table 3). Sensitivity analysis with exclusion of open cases from both cohorts demonstrated loss of significance in serious complications, but an increase in odds of 30-day mortality (OR 53) suggestive of concordance.

**Table 3:** Multivariable logistic regression for predictors of serious complications and 30-day mortality

Variable	Serious Complications OR (95% CI), *P*-value	30-Day Mortality OR (95% CI), *P*-value
Age (per 10 years)	1.17 (1.15–1.19), *p* < 0.001	1.73 (1.55–1.93), *p* < 0.001
Female sex	0.91 (0.87–0.95), *p* < 0.001	0.53 (0.42–0.67), *p* < 0.001
Body mass index (per 5 kg/m²)	1.00 (0.98–1.01), *p* = 0.45	1.30 (1.24–1.37), *p* < 0.001
Diabetes (non-insulin)	1.00 (0.95–1.05), *p* = 0.93	1.17 (0.88–1.55), *p* = 0.29
Diabetes (insulin-dependent)	1.22 (1.14–1.31), *p* < 0.001	1.94 (1.42–2.67), *p* < 0.001
Hypertension	1.19 (1.14–1.24), *p* < 0.001	1.35 (1.02–1.79), *p* = 0.033
Renal insufficiency	2.07 (1.79–2.41), *p* < 0.001	2.31 (1.32–4.03), *p* = 0.003
History of myocardial infarction	1.52 (1.34–1.72), *p* < 0.001	1.82 (1.13–2.94), *p* = 0.014
Therapeutic anticoagulation	1.99 (1.85–2.14), *p* < 0.001	2.52 (1.85–3.43), *p* < 0.001
Albumin (per 1 g/dL increase)	0.69 (0.66–0.72), *p* < 0.001	0.60 (0.46–0.78), *p* < 0.001
Simultaneous kidney transplantation	7.97 (2.27–27.93), *p* = 0.001	33.99 (3.99–289.03), *p* = 0.001

## Discussion

This study represents the largest known multi-institutional analysis of patients undergoing simultaneous SG + KT, leveraging four years of data from the MBSAQIP registry. Although rare, comprising just 22 cases among over 582,000 sleeve gastrectomies (< 0.01%), SG + KT was associated with dramatically increased rates of serious complications, readmission, bleeding, venous thromboembolism, and 30-day mortality, even after adjustment for comorbidities. The higher rate of “serious complications” compared to individual complications reflect the composite nature of this outcome that are not individually reported.

The significantly higher bleeding rates, readmissions, and serious complications in the SG + KT cohort reflect the unique challenges of performing SG in the setting of ESRD and kidney transplant. ESRD patients often present with uremic coagulopathy, which can cause platelet dysfunction and anemia and contribute to an increased bleeding risk [11]. We recommend multimodal approaches to minimize bleeding risk, such as staple line reinforcements, tranexamic acid when appropriate and the liberal use of hemostatic adjuncts.

Furthermore, hypoalbuminemia in this cohort likely reflects the complex metabolic and inflammatory state associated with ESRD. Reduced albumin levels are associated with impaired collagen synthesis, wound healing, and decreased oncotic pressure, potentially increasing susceptibility to postoperative complications [12]. Additionally, concurrent immunosuppression medications also interfere with wound healing and increase susceptibility to infections [13]. This could potentially explain the higher readmission rates observed.

Beyond these concerns, SG + KT presents additional challenges. The restrictive nature of SG can limit oral intake early in the postoperative period, potentially leading to dehydration, which is particularly concerning in newly transplanted kidneys that require adequate hydration for optimal function [14]. Immunosuppression protocols following transplantation may be influenced by the altered gastrointestinal physiology and anatomy after SG, potentially affecting drug clearance, systemic exposure, and pharmacodynamics. Close monitoring of immunosuppressive levels will therefore be essential to avoid overdosing and potential side effects [15]. To address these challenges, we recommend a modified immunosuppression protocol with close therapeutic drug monitoring and continued research on how bariatric surgery impacts absorption of these medications. We also recommend aggressive IV fluid resuscitation guided by hemodynamic parameters and urine output rather than standard bariatric protocols, including outpatient rehydration therapy.

However, our findings challenge the assumption that outcomes are comparable to staged approaches, particularly in the broader, multi-institutional setting. The observed 30-day mortality rate of 9.1% in SG + KT patients, over 90-fold higher than SG alone, reflects the cumulative burden of dual procedures. Regarding KT alone, recent analyses of large national cohorts indicate that early post-transplant mortality has declined over the past two decades, reflecting improvements in perioperative care, immunosuppression, and patient selection with a documented treatment-related mortality of 0.5% at 30-days and 1% at 3 months [16]. A large US cohort study reported a 30-day post-transplant mortality rate of 2.6% for death and 2.9% for acute myocardial infarction [17]. Kidney transplantation in patients with obesity showed inferior early kidney graft function but no difference at long-term follow-up. However, recipient obesity demonstrated a negative effect on patient survival and postoperative complications [18]. 

Our multivariable analysis identified simultaneous KT as the strongest independent predictor of both serious complications and mortality, with an odds ratio of nearly 34 for death. This finding persisted despite adjustment for age, BMI, albumin, renal function, and cardiovascular risk factors, highlighting the profound impact of combining two major operations in a single setting.

There is growing interest in optimizing transplant candidacy for patients with obesity, and bariatric surgery, particularly sleeve gastrectomy, has emerged as a leading strategy. Previous studies have shown that SG can safely facilitate weight loss, improve comorbidities, and enhance access to kidney transplantation. However, the timing of SG relative to KT remains debated. Staged approaches allow for preoperative optimization but introduce delays that may lead to worsening renal function or missed donor opportunities. Simultaneous SG + KT theoretically mitigates this but appears to carry a substantial increase in perioperative risk.

This analysis has important clinical implications. Patient selection is paramount, and simultaneous SG + KT should likely be reserved for highly selected individuals where benefits clearly outweigh risks. Moreover, high-volume centers with extensive experience in both SG and KT may be better positioned to manage such cases. Our results align with prior single-center studies and case series suggesting that SG + KT is feasible but technically demanding. Spaggiari et al. demonstrated the potential of robotic-assisted simultaneous SG + KT, emphasizing benefits in select high-volume centers with multidisciplinary expertise [19]. 

This study is limited by its retrospective design and reliance on administrative coding within the MBSAQIP database. The data set does not distinguish between planned open procedures and conversions from minimally invasive approaches, limiting interpretation of the higher rate of open surgery in the SG + KT cohort. It also lacks transplant-specific variables such as donor type (living vs. deceased), graft characteristics, immunosuppressive regimens, and center- or surgeon-level factors including volume, experience, or operative technique. MBSAQIP does not capture transplant waitlist status or eligibility, limiting the comparison of transplant eligible ESRD patients undergoing SG alone, hence the comparison to available proxies like dialysis-dependent patients. Additionally, long-term outcomes such as graft function, metabolic improvements, and renal durability could not be assessed. The relatively modest performance of the serious complication regression model demonstrated loss of significance upon exclusion of open cases within sensitivity analysis, emphasizing the underpowered nature of this study and the challenging heterogeneity of the study outcome. The rarity of simultaneous SG + KT in the dataset thus limits statistical power for subgroup analyses and may affect generalizability. Nonetheless, the dataset’s breadth provides valuable insight into national trends and early outcomes associated with this evolving concept. Given the markedly elevated risk of complications and mortality, SG + KT should be approached with significant caution after rigorous patient selection where comprehensive informed consent is critical. These patients should be managed within multidisciplinary teams that include transplant surgery, bariatric surgery, nephrology, nutrition, and perioperative care specialists. Until more robust data are available, simultaneous procedures should likely be reserved for highly selected patients in experienced, high-volume centers. Future research should focus on identifying predictors of favorable outcomes, establishing standardized care pathways, and evaluating whether outcomes can be improved through careful patient selection or institutional expertise.

## Conclusion

Of 582,860 patients who underwent SG from 2020 to 2023 in the MBSAQIP database, only 22 patients had simultaneous KT. SG + KT was associated with substantially increased 30-day morbidity and mortality, and multivariable regression identified that simultaneous KT was the single greatest predictor of mortality (OR 33.99) after adjusting for comorbidities. Given the magnitude of this perioperative risk, these findings support staged approaches to bariatric surgery and kidney transplantation as the safer alternative. When simultaneous SG + KT is considered, it should be performed only in highly selected patients at experienced, high-volume centers, under strict institutional review protocols, and with comprehensive informed consent that clearly communicates the elevated mortality risk relative to staged procedures. Prospective studies and multi-center registries capturing bariatric-transplant cohorts are needed to refine patient selection and determine whether institutional factors can mitigate these risks.

## Data Availability

No datasets were generated or analysed during the current study.

## Declarations

### Prior Presentation

This work has been presented at the 2025 American Society for Metabolic and Bariatric Surgery (ASMBS) Annual Meeting.

## References

[1] Ward ZJ, Bleich SN, Cradock AL, et al. Projected U.S. state-level prevalence of adult obesity and severe obesity. N Engl J Med. 2019;381(25):2440–50. 10.1056/NEJMsa1909301. 31851800 10.1056/NEJMsa1909301

[2] Veroux M, Mattone E, Cavallo M, et al. Obesity and bariatric surgery in kidney transplantation: a clinical review. World J Diabetes. 2021;12(9):1563–75. 10.4239/wjd.v12.i9.1563. 34630908 10.4239/wjd.v12.i9.1563 PMC8472502

[3] Yun HR, Kim H, Park JT, Chang TI, et al. Obesity, metabolic abnormality, and progression of CKD. Am J Kidney Dis. 2018;72(3):400–10. 10.1053/j.ajkd.2018.02.362. 29728317 10.1053/j.ajkd.2018.02.362

[4] Chadban SJ, Ahn C, Axelrod DA, et al. KDIGO clinical practice guideline on the evaluation and management of candidates for kidney transplantation. Transplantation. 2020;104(4S1 Suppl 1):S11–103. 10.1097/TP.0000000000003136. 32301874 10.1097/TP.0000000000003136

[5] Kassam AF, Mirza A, Kim Y, et al. Long-term outcomes in patients with obesity and renal disease after sleeve gastrectomy. Am J Transplant. 2020;20(2):422–9. 10.1111/ajt.15650. 31605562 10.1111/ajt.15650

[6] Kim Y, Jung AD, Dhar VK, et al. Laparoscopic sleeve gastrectomy improves renal transplant candidacy and posttransplant outcomes in morbidly obese patients. Am J Transplant. 2018;18(2):410–6. 10.1111/ajt.14463. 28805345 10.1111/ajt.14463

[7] Zaminpeyma R, Claus M, Paraskevas S, Court O, Tchervenkov J, Andalib A. Outcomes of kidney transplant recipients who underwent pre-transplant bariatric surgery for severe obesity: a long-term follow-up study. Surg Endosc. 2023;37(1):494–502. 10.1007/s00464-022-09552-9. 36002684 10.1007/s00464-022-09552-9 PMC9401197

[8] Pané A, Molina-Andujar A, Olbeyra R, et al. Bariatric surgery outcomes in patients with kidney transplantation. J Clin Med. 2022;11(20):6030. 10.3390/jcm11206030. 36294351 10.3390/jcm11206030 PMC9604744

[9] Guggino J, Coumes S, Wion N, Reche F, Arvieux C, Borel AL. Effectiveness and safety of bariatric surgery in patients with end-stage chronic kidney disease or kidney transplant. Obesity (Silver Spring). 2020;28(12):2290–304. 10.1002/oby.23001. 33230959 10.1002/oby.23001

[10] Spaggiari M, Martinino A, Bencini G, et al. Timing considerations for sleeve gastrectomy in kidney transplant patients: a single center evaluation. Transpl Int. 2024;37:12690. 10.3389/ti.2024.12690. 38957660 10.3389/ti.2024.12690 PMC11217181

[11] Baaten CCFMJ, Sternkopf M, Henning T, Marx N, Jankowski J, Noels H. Platelet function in CKD: a systematic review and meta-analysis. J Am Soc Nephrol. 2021;32(7):1583–98. 10.1681/ASN.2020101440. 33941607 10.1681/ASN.2020101440 PMC8425648

[12] McLean C, Mocanu V, Birch DW, Karmali S, Switzer NJ. Hypoalbuminemia predicts serious complications following elective bariatric surgery. Obes Surg. 2021;31(10):4519–27. 10.1007/s11695-021-05641-1. 34378157 10.1007/s11695-021-05641-1

[13] Appoo A, Christensen BL, Somayaji R. Examining the association between immunosuppressants and wound healing: a narrative review. Adv Skin Wound Care. 2024;37(5):261–7. 10.1097/ASW.0000000000000127. 38648239 10.1097/ASW.0000000000000127

[14] Yamanaga S, Hidaka Y, Kawabata C, et al. Water intake, baseline biopsy, and graft function after living donor kidney transplantation. Sci Rep. 2024;14(1):3715. 10.1038/s41598-024-54163-0. 38355944 10.1038/s41598-024-54163-0 PMC10866883

[15] Chan G, Hajjar R, Boutin L, Garneau PY, et al. Prospective study of the changes in pharmacokinetics of immunosuppressive medications after laparoscopic sleeve gastrectomy. Am J Transplant. 2020;20(2):582–8. 10.1111/ajt.15602. 31529773 10.1111/ajt.15602

[16] Choi H, Lee W, Lee HS, et al. The risk factors associated with treatment-related mortality in 16,073 kidney transplantation-A nationwide cohort study. PLoS One. 2020;15(7):e0236274. 10.1371/journal.pone.0236274. 32722695 10.1371/journal.pone.0236274 PMC7386583

[17] Cheng XS, Liu S, Han J, et al. Association of pretransplant coronary heart disease testing with early kidney transplant outcomes. JAMA Intern Med. 2023;183(2):134–41. 10.1001/jamainternmed.2022.6069. 36595271 10.1001/jamainternmed.2022.6069 PMC9857067

[18] Buemi A, Romero L, Zech F, et al. Impact of recipient obesity on kidney transplantation outcome: a retrospective cohort study with a matched comparison. Transplant Proc. 2022;54(7):1786–94. 10.1016/j.transproceed.2022.03.058. 35940948 10.1016/j.transproceed.2022.03.058

[19] Spaggiari M, Di Cocco P, Tulla K, et al. Simultaneous robotic kidney transplantation and bariatric surgery for morbidly obese patients with end-stage renal failure. Am J Transplant. 2021;21(4):1525–34. 10.1111/ajt.16322. 32976702 10.1111/ajt.16322

